# TGF-ß Sma/Mab Signaling Mutations Uncouple Reproductive Aging from Somatic Aging

**DOI:** 10.1371/journal.pgen.1000789

**Published:** 2009-12-24

**Authors:** Shijing Luo, Wendy M. Shaw, Jasmine Ashraf, Coleen T. Murphy

**Affiliations:** Lewis-Sigler Institute for Integrative Genomics and Department of Molecular Biology, Princeton University, Princeton, New Jersey, United States of America; Stanford University Medical Center, United States of America

## Abstract

Female reproductive cessation is one of the earliest age-related declines humans experience, occurring in mid-adulthood. Similarly, *Caenorhabditis elegans*' reproductive span is short relative to its total life span, with reproduction ceasing about a third into its 15–20 day adulthood. All of the known mutations and treatments that extend *C. elegans*' reproductive period also regulate longevity, suggesting that reproductive span is normally linked to life span. *C. elegans* has two canonical TGF-ß signaling pathways. We recently found that the TGF-ß Dauer pathway regulates longevity through the Insulin/IGF-1 Signaling (IIS) pathway; here we show that this pathway has a moderate effect on reproductive span. By contrast, TGF-ß Sma/Mab signaling mutants exhibit a substantially extended reproductive period, more than doubling reproductive span in some cases. Sma/Mab mutations extend reproductive span disproportionately to life span and act independently of known regulators of somatic aging, such as Insulin/IGF-1 Signaling and Dietary Restriction. This is the first discovery of a pathway that regulates reproductive span independently of longevity and the first identification of the TGF-ß Sma/Mab pathway as a regulator of reproductive aging. Our results suggest that longevity and reproductive span regulation can be uncoupled, although they appear to normally be linked through regulatory pathways.

## Author Summary

Female reproductive cessation is the earliest aging phenotype humans experience, and its importance as a clinical issue is growing as more women opt to have children later in life. While much work has been done to understand the general aging process, little is currently known about the regulation of reproductive aging. Like longevity, the ability to produce progeny with advanced age is likely to be genetically regulated. Thus, understanding the processes that regulate reproductive aging may allow us to address the problems of maternal age-related infertility and birth defects. *C. elegans* and humans both have long post-reproductive life spans, leaving open the possibility that their reproductive spans might be extendable. *C. elegans* has been used previously to discover conserved regulators of aging, and here we use worms to identify a new regulator of reproductive aging, a highly conserved TGF-ß signaling pathway. We find that TGF-ß signaling regulates reproductive aging independently of somatic aging. This is the first identification of a pathway that breaks the coupling that normally links the two processes. Our work will provide new insights into the improvement of human fertility and prevention of age-related birth defects, and it has implications for the evolutionary relationship between reproduction and longevity regulation.

## Introduction

Among human age-related declines, female reproductive cessation is one of the earliest to occur, with infertility and maternal age-related birth defects arising during the fourth decade of life [1]. While artificial reproductive technologies have improved late conception success [2]–[5], the underlying molecular regulators of reproductive cessation remain largely unknown. Like longevity, the ability to produce progeny with advanced age is likely to be genetically regulated. Thus, understanding the processes that regulate reproductive aging may allow us to address the problems of maternal age-related infertility and birth defects.

Although *C. elegans* produces large broods of progeny and does not care for its young, its reproductive schedule is similar to human females' in that its fertility and reproduction sharply decline in early/mid-adulthood, followed by a long post-reproductive period [6],[7]. The similarities between *C. elegans* and human reproductive schedules suggest the intriguing possibility that studies in this model organism may reveal mechanisms regulating reproductive cessation across species.

While many *C. elegans* studies have focused on reproductive fitness, measuring total numbers of offspring produced and average generation time, we are instead interested in identifying regulators of late-life reproduction (i.e., the ability of individual mothers to continue to reproduce viable progeny as they age). The standard assessments of fitness and fertility do not ascertain the length of time that an individual is capable of successful reproduction. However, we and others [6]–[8] have recently become interested in the latter aspect of reproduction, because of the obvious possible parallels with human reproductive aging, in particular, Advanced Maternal Age (AMA) and its related clinical problems. In other words, these model organism studies of reproductive aging are focused on determining the capacity to reproduce successfully late in life, rather than on total progeny production or evolutionary fitness. Hughes, et al. recently showed that worms undergo reproductive aging, a process that is dependent neither on tissue wear (as manipulation of early progeny number had no influence) nor on sperm availability [7]. Thus, the reproductive system of *C. elegans* ages significantly during the first week of adulthood, which is also reflected in the degree of germ line degeneration and oocyte quality decline [8],[9]. This germ line aging results in reproductive cessation days to weeks prior to death and a relatively long post-reproductive life span, similar to human females' long post-reproductive life span.

The *C. elegans* mutants currently known to delay reproductive aging were originally identified through their longevity phenotypes [7]. These longevity mutants include the insulin/IGF-1 receptor mutant *daf-2* [7],[10],[11], and a model of Dietary Restriction (DR), *eat-2* [6],[12]. Insulin/IGF-1 signaling (IIS) and FOXO transcription factor activity have been implicated in the regulation of reproduction in several other organisms, including *Drosophila* [13], mice [14],[15], and humans [16]. Life span extension and slowing of reproductive activity are also hallmarks of Dietary Restriction. Dietary Restriction reduces progeny number and lengthens the reproductive period of *C. elegans* hermaphrodites [7], female *Drosophila* [16], and female rodents [18],[19]. *C. elegans* shifts its reproductive strategy after starvation, modifying its production of males and its outcrossing frequency [17], and many animals adjust their reproductive life span in response to predation levels [18]. Together, these data indicate that the reproductive schedule is flexible, poised to respond to environmental and molecular perturbations, and that the mechanisms regulating reproductive aging, like longevity, are likely to be evolutionarily conserved.

**Figure 1:**
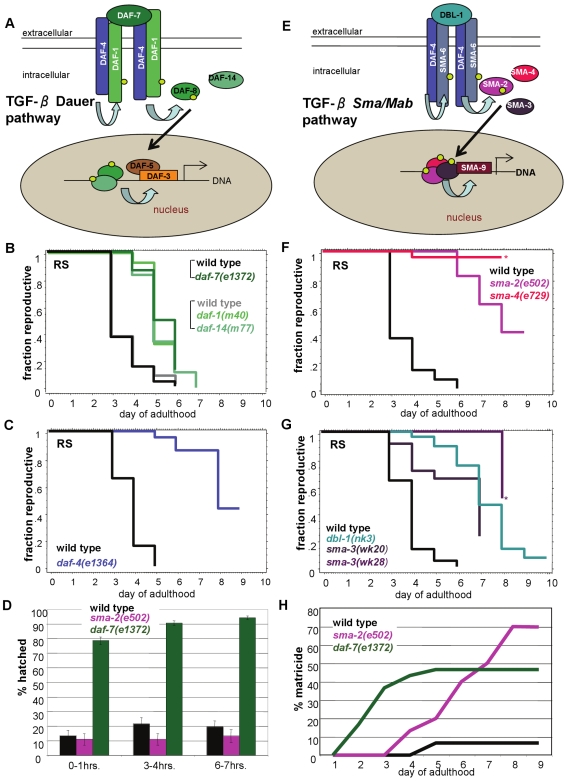
TGF-ß Sma/Mab pathway regulates reproductive span. (A, E) Schematic representation of the TGF-ß Dauer and TGF-ß Sma/Mab pathways in *C. elegans*. (B) Mutants of the TGF-ß Dauer pathway moderately extend reproductive span (two experiments are shown). (C) A mutant of the shared receptor between the two pathways, *daf-4*, doubles the reproductive span of wild type. (D) *daf-7*, but not *sma-2*, animals lay more-developed progeny than wild type: percentage of embryos hatched 0–1, 3–4, and 6–7 hours after laying from day 3 wild-type, *daf-7* and *sma-2* adults. (F, G) Mutants of the TGF-ß Sma/Mab pathway greatly extend reproductive span. (H) Cumulative percentage of matricide animals (caused by internal progeny hatching, same below). *daf-7*'s matricide rate is high starting in early adulthood, while *sma-2*'s increases with age, finally resulting in more matricide animals. All reproductive spans in this figure are measured in self-fertilized hermaphrodites. Asterisk indicates high matricide frequency. Additional statistics presented in Table S1 and Table S2.

*C. elegans* has two highly conserved Transforming Growth Factor-ß (TGF-ß) signaling pathways, the Dauer (*daf-7*) and Sma/Mab (*dbl-1*) pathways. We recently found that TGF-ß Dauer signaling regulates lifespan through its interactions with the Insulin/IGF-1 Signaling (IIS) pathway [19]. Members of the TGF-ß Dauer pathway include the ligand DAF-7, receptor heterodimers DAF-1 and DAF-4, the R-Smads (receptor-regulated Smad signal transducer) DAF-8 and DAF-14, the Co-Smad (common-mediator Smad) DAF-3, and the transcription factor DAF-5 (Figure 1A; mammalian homologs are shown in Figure S1A) [20],[21]. DAF-4 is a type II receptor that is shared between the Dauer pathway and a second TGF-ß pathway, the Sma/Mab pathway (Small body/Male tail abnormal). The Sma/Mab pathway includes the ligand DBL-1, the type I receptor SMA-6, SMA-2 and SMA-3 R-Smads, the Co-Smad SMA-4, and the SMA-9 transcription co-factor (Figure 1E; mammalian homologs shown in Figure S2A) [22]–[25].

Here we show that the Dauer pathway has a moderate effect on reproductive span, mediated at least in part by insulin/FOXO activity. More importantly, we have found that its shared member with the TGF-ß Sma/Mab pathway, *daf-4*, and the entire TGF-ß Sma/Mab pathway, strongly influence reproductive aging. Reduced TGF-ß Sma/Mab signaling extends reproductive span disproportionately to life span, and is genetically independent of known longevity regulators. The TGF-ß Sma/Mab pathway is a novel regulator of reproductive aging, and the first regulator of reproductive aging to be identified independently of somatic aging regulation. Our results demonstrate that somatic aging and reproductive aging can be uncoupled, suggesting that different molecular mechanisms underlie the two processes, but may normally be linked.

## Results

### DAF-4, a shared TGF-ß co-receptor, regulates reproductive aging

In addition to its regulation of dauer formation [26], we recently found that the TGF-ß Dauer pathway (Figure 1A, Figure S1A) regulates longevity [19]. However, whether this pathway also plays a role in the regulation of reproductive aging is unknown. To analyze the effect of TGF-ß Dauer mutants on reproduction, we determined the proportion of adults capable of progeny production as a function of age. The “reproductive span” calculated from such assays (see Materials and Methods) reflects the period of time animals produce viable progeny, as described previously [6],[7]. We found that members of the TGF-ß Dauer pathway moderately extended reproduction (Figure 1B; Figure S1B, S1C): while wild type's mean reproductive span was ∼3.5 days, the means of *daf-7*, *daf-1*, *daf-8*, and *daf-14* mutants were 4–5 days, extensions of 25–50% (Table S2; Figure S1D). In addition, their maximum reproductive spans were ∼1 day longer than wild type's. At least part of the moderate reproductive span extension is likely a result of delayed onset of reproduction due to an egg-laying (Egl) defect (Figure 1D) [19],[27],[28]; by the end of *daf-7's* reproductive span, many progeny hatched into L1 larvae immediately upon being laid, as opposed to the typical 12–16 hour hatching time of wild-type eggs. *daf-7* progeny do not develop into adults faster than wild type, so the advanced developmental stage of the progeny is likely due to egg retention in the mother.

Unlike the moderate reproductive span extensions of other TGF-ß Dauer pathway mutants, *daf-4's* ∼8 day mean is more than double the reproductive span of wild-type animals (Figure 1C; Figure S1D; Table S2). *daf-4* mutants continued to steadily produce progeny for several days after reproductive cessation in wild-type animals, and its maximum reproductive span was 4–5 days longer than wild type (Figure S1G). This dramatic difference cannot be explained by the egg-laying defect typical of the TGF-ß Dauer pathway mutants, which extends reproductive span a maximum of one day. *daf-4* encodes *C. elegans'* sole ortholog of the type II TGF-ß co-receptor, and is utilized by both the Dauer pathway and a second TGF-ß pathway, the Sma/Mab pathway (Figure 1E, Figure S2A) [22]–[25]. The large reproductive span extension that we observed in *daf-4* animals, but not other TGF-ß Dauer mutants, suggested the possibility that the Sma/Mab pathway might be important in the regulation of reproductive aging.

### TGF-ß Sma/Mab signaling regulates reproductive aging

We measured the reproductive spans of seven alleles of TGF-ß Sma/Mab pathway mutants (Figure 1E, Figure S2A), and found that decreased TGF-ß Sma/Mab signaling indeed increased reproductive span significantly: similar to *daf-4*, the mean reproductive spans of *sma-2* and *dbl-1* were over 7 days, compared to ∼3.5 days in wild type; the rest of the mutants in this pathway (*sma-3*, *sma-4*, *sma-6*, and *sma-9*) also increased reproductive span substantially (Figure 1F and 1G; Table S1; Figure S2B, S2C). The hatching rates of Sma/Mab mutants were comparable to wild type (Figure 1D), the onset of progeny production was not delayed, and progeny were steadily produced beyond the age when wild-type reproduction ceased (Figure S3A, S3B, S3C). Similar to *daf-2* and *eat-2*, mutants that also extend reproductive span [7], Sma/Mab mutants produce fewer total progeny over a longer period of time (Table S3; Figure S3). The reproductive span extensions and progeny production profiles of the Sma/Mab mutants contrast with the delayed onset and sharp decline in the number of progeny produced after peak reproduction by the TGF-ß Dauer mutants (Figure S1E, S1F), suggesting that the Dauer and Sma/Mab mutants are distinct in their reproductive aging phenotypes.

Sma/Mab mutants exhibited a highly penetrant late egg-laying defect and internal hatching (matricide, or “bagging”) at the end of their reproductive period, in contrast to the Dauer mutants' very early egg-laying (Egl) and bagging phenotypes (Figure 1H, Figure S2E). In fact, several assays were terminated when a large fraction of the worms were still reproductive, due to the Sma/Mab mutants' late matricide phenotype (see asterisked *sma-4* in Figure 1F and *sma-3* in Figure 1G). It is likely that the full late reproductive capacity of the Sma/Mab mutants is masked by this late matricide defect. Thus, while the TGF-ß Dauer mutants' delayed onset of reproduction and Egl phenotypes may account for part of their moderate reproductive span increases (a maximum of one day), these factors are not likely the cause of TGF-ß Sma/Mab mutants' dramatic reproductive span extensions.

### Sma/Mab reproductive span extension is independent of sperm contribution

**Figure 2:**
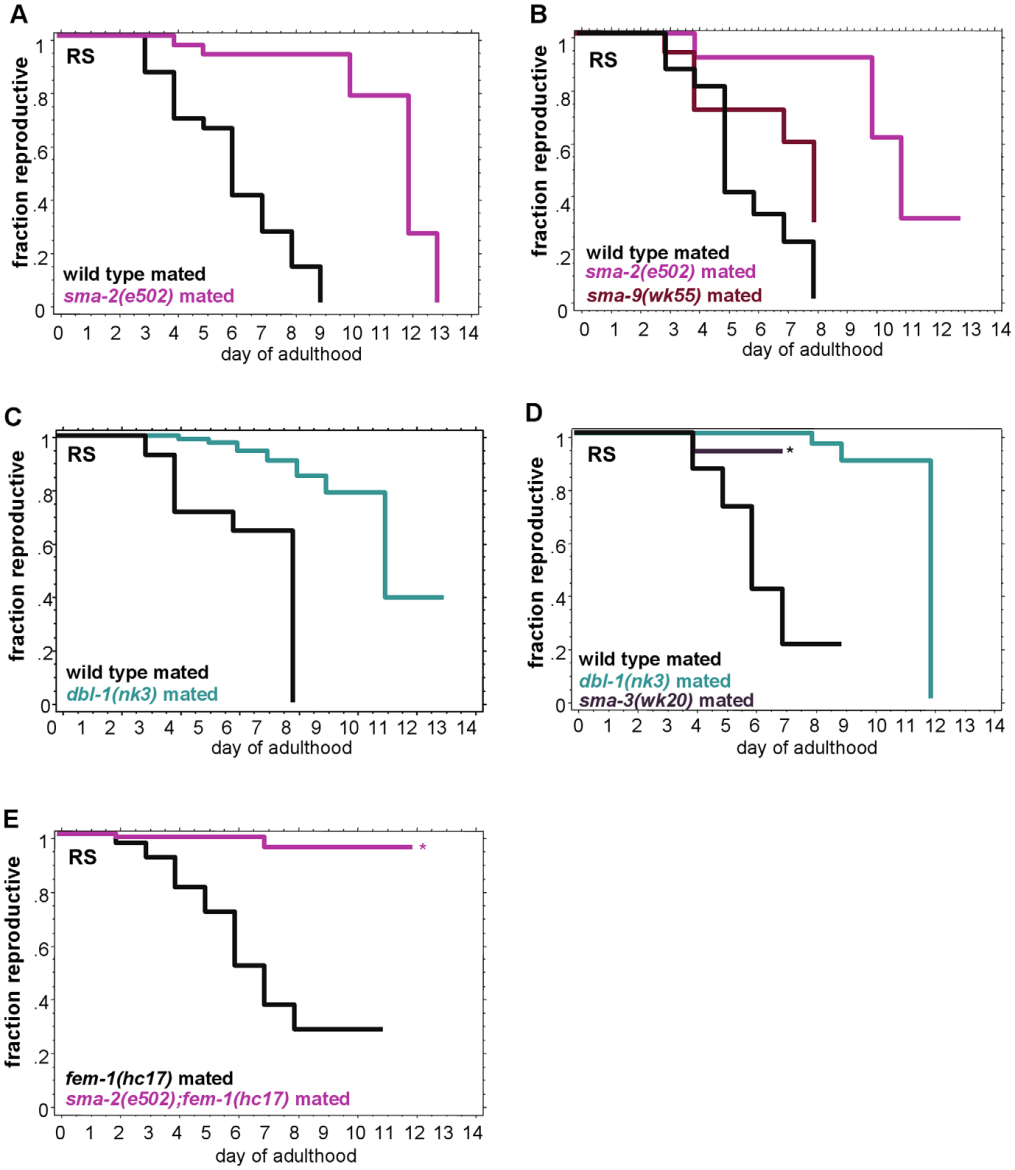
TGF-ß Sma/Mab mutant reproductive span extensions are independent of sperm contribution. (A–D) TGF-ß Sma/Mab mutants mated with young wild-type males have significantly longer reproductive spans than mated wild-type animals. (E) After mating with wild-type males, *sma-2;fem-1* (spermless) animals also have greater reproductive spans than *fem-1* worms. Asterisk indicates a high matricide frequency. Additional statistics presented in Table S1.

*C. elegans* hermaphrodite sperm number limits wild-type self-fertilized reproduction, but mating with young (day 1 adult) males, whose sperm are not limited and outcompete those of the hermaphrodite, increases and prolongs progeny production [29]–[31]. To rule out the possibility that the reproductive span extensions we observed in TGF-ß Sma/Mab mutants are due to increased or extended sperm production or utilization, we mated Sma/Mab mutant hermaphrodites with young wild-type males. We found that Sma/Mab mutants significantly and consistently increased mated reproductive span, from wild type's mean mated reproductive span of ∼6.0 days to a mean of 10–11 days (Figure 2A–2D; Table S1). In fact, Sma/Mab mutants were usually still fertile through Day 12–13 of adulthood, compared to the complete cessation of reproduction by Day 8–9 in wild-type animals. (Interestingly, when mated with wild-type males at an older age (day 4), the Sma/Mab mutants still had significant reproductive span extensions (Figure S2D), further supporting the notion that sperm quality and number are not the limiting factor, as shown previously [7].)

To further eliminate the possibility of sperm contribution, we also tested feminized (*fem-1*) mutant hermaphrodites, which fail to make sperm when raised at restrictive temperatures. *fem-1* mutants mated with wild-type males have a mean reproductive span of 6.3 days, while more than 90% of the mated *sma-2;fem-1* double mutants were still fertile at day 12 (Figure 2E). Notably, the self-fertilized reproductive spans of Sma/Mab mutants are even longer than wild type's mated reproductive span, highlighting the extreme extensions shown by Sma/Mab mutants (compare Figure 1F and Figure 2; Table S1). Additionally, neither the self-fertilized nor the mated Sma/Mab mutants delay the onset of progeny production, and both continue to produce progeny steadily beyond the age of wild-type reproductive cessation (Figure S3).

In self-fertilized worms, sperm is only made prior to oogenesis [24], and in mated worms sperm is in excess, thus the extended reproductive span we observed cannot be due to extended spermatogenesis. Our results, together with the Hughes, et al. data, suggest that significant reproductive aging already occurs prior to the cessation of sperm availability in self-fertilized animals, and that Sma/Mab mutants, like IIS (*daf-2*) and DR (*eat-2*) mutants [7], slow the rate of aging of the reproductive system.

### Reproductive span extension is independent of body size, ovulation rate, early progeny number, and brood size

We noticed that the Sma/Mab mutants produced fewer progeny than wild type each day in the early phase of reproduction (Figure S3; Figure S4D, S4E, S4F), and have smaller broods (Table S3, Table S4). This reduction in progeny number reflects slower ovulation rate of the mutants in early reproduction (Figure S4C), likely due to their small body size (Figure S4A, S4B). In fact, it has been suggested that reduction of *C. elegans* progeny number is linked to small body size via physical constraint of the maternal gonad and/or body size [32]–[35]. The downstream transcription co-factor of the Sma/Mab pathway, *sma-9*, is required in early larval development for the regulation of body size before gametogenesis [25],[36]; however, we find that reduction of *sma-9* only in adulthood is sufficient to extend late reproduction (Figure S5), suggesting that the growth and reproductive aging functions of the Sma/Mab pathway are independent.

**Figure 3:**
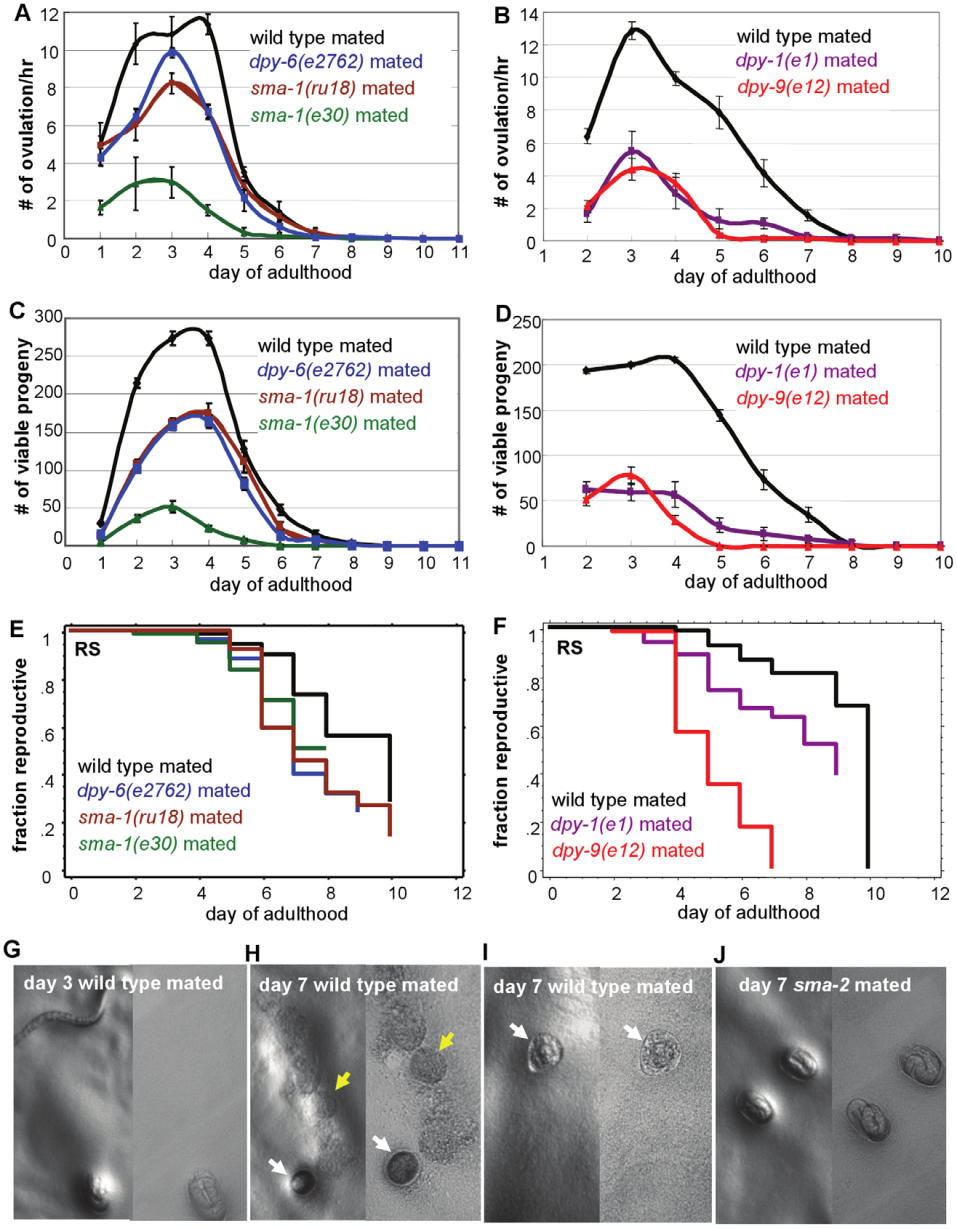
Reproductive span is independent of body size, ovulation rate, and progeny number. Mated non-TGF-ß small mutants all have slower ovulation rates (A, B), and produce fewer early and total progeny (C, D) than wild type. (E, F) Mated reproductive spans of the non-TGF-ß small mutants are not longer than wild type. Additional statistics presented in Table S5. (G–J) Example images of viable embryos (G, J), unhatchable embryos (H, I, white arrows), and unfertilized oocytes (H, yellow arrows) laid by wild type (G–I) or *sma-2* (J) mated animals. Images on the left were taken with Leica PLANAPO 1.6× objective, on the right with PLANAPO 5×.

In mated assays, sperm number is not limiting; therefore, one possible explanation for extended reproductive span of the Sma/Mab mutants is that oocyte number is limiting, and thus slower ovulation allows the mutants to use up their oocyte supply more slowly. To test this hypothesis as well as the body size effect on reproduction, we examined five small but non-TGF-ß mutants (*dpy-6(e2762)*, *sma-1(ru18)*, *sma-1(e30)*, *dpy-1(e1)* and *dpy-9(e12))* whose body sizes are similar to the TGF-ß Sma/Mab mutants (compare Figure S4A and S4B and Figure S6A and S6B). Importantly, none of these strains have been reported to have egg-laying or embryonic developmental abnormalities or effects on longevity, and therefore serve as a fair set of samples for comparison. As expected, the five small mutants have slower ovulation rates (Figure 3A and 3B), and as a result produce fewer early progeny and fewer total progeny (Figure 3C and 3D). However, unlike the TGF-ß mutants, none of the small, non-TGF-ß mutants extended mated reproductive span (Figure 3E and 3F). In fact, all of the mutants had shorter reproductive spans. These data suggest that small body size and reductions in ovulation rate and progeny number do not increase reproductive span, but rather are usually associated with shorter reproductive spans. Therefore the TGF-ß Sma/Mab mutants are special in their extension of reproductive span.

We also addressed whether oocyte number becomes the limiting factor when sperm is no longer limiting by mating animals with young wild-type males, which is one basis for the assumption that slow ovulation extends reproductive span. On day 3, all wild-type animals (n = 12) produced only eggs that were able to hatch and develop to viable progeny (Figure 3G), but on day 7, 59% (n = 17) of the animals laid oocytes that failed to be fertilized (Figure 3H) and/or eggs that were unable to hatch (Figure 3H and 3I), resulting in cessation of viable progeny production. Therefore, the limiting factor is not number of oocytes, which are clearly still in excess, but the quality of the oocytes. By contrast, the majority of *sma-2* mutants still produced exclusively viable eggs on day 7 (Figure 3J), with only 19% (n = 16, p = 0.03 compared with wild type) of the animals starting to lay unfertilized oocytes or unhatchable eggs. Our data, together with the observation that late reproduction is independent of early reproduction [7], suggest that Sma/Mab mutants extend reproductive span independently of body size, ovulation rate, early progeny number, and brood size, but instead by improving oocyte quality.

### Sma/Mab signaling regulates reproductive span disproportionately to life span

**Figure 4:**
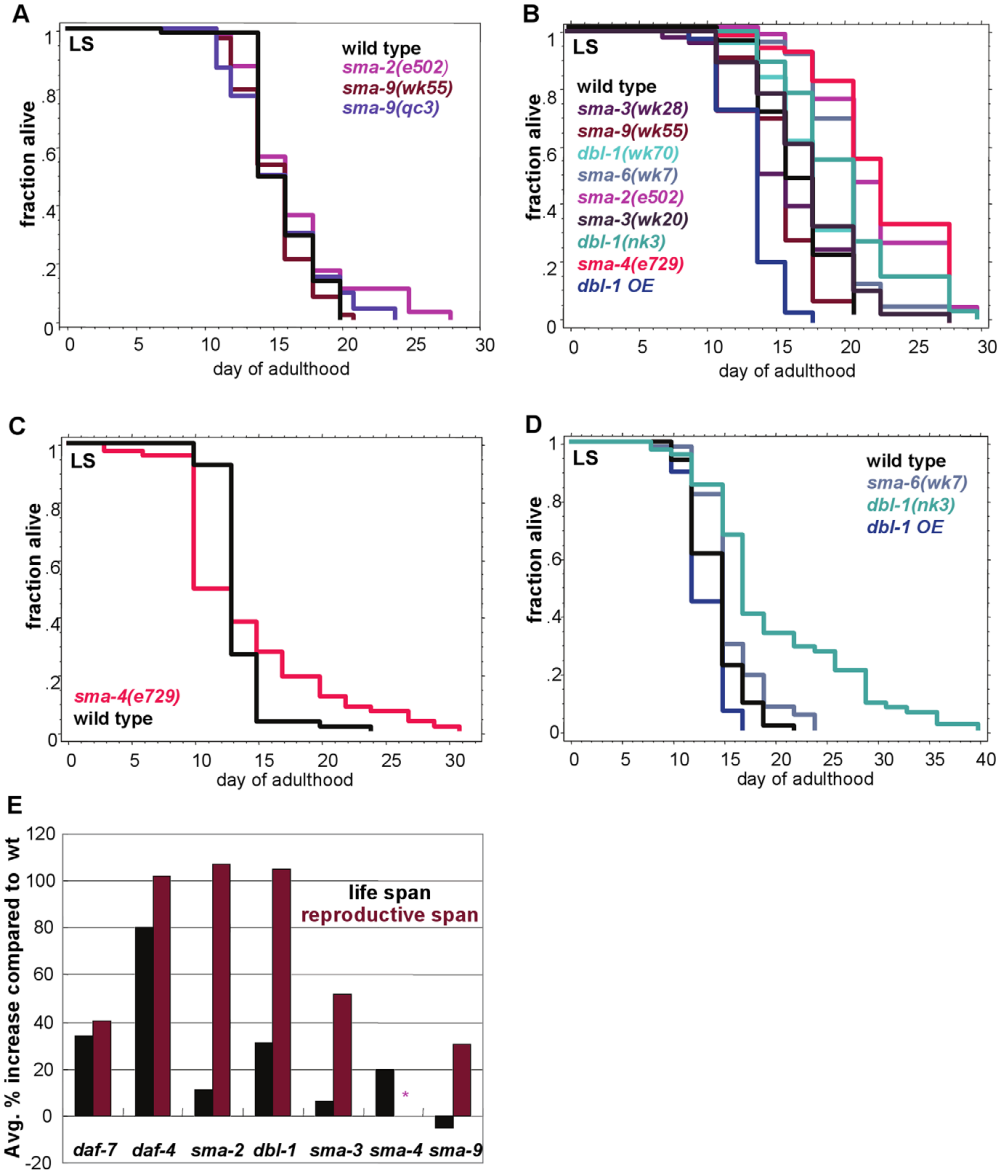
TGF-ß Sma/Mab mutants have larger effects on reproductive span than on life span. (A–D) Life span assay trials. *dbl-1* and *sma-6* mutants increase life span moderately (B, D), *sma-2* and *sma-4* mutants have inconsistent effects on life span (A–C), and *sma-3* and *sma-9* appear to have no effect on longevity (A, B). *dbl-1 OE* (over-expression) slightly shortens life span (B, D). (Additional statistics presented in Table S6.) (E) Average percent change from wild-type life span and reproductive span of TGF-ß Dauer and Sma/Mab mutants. Sma/Mab mutants all have larger effects on reproductive span than on life span. Asterisk indicates that the value is not available due to high matricide rate.

While *daf-2* and *eat-2* regulate reproductive aging [6],[7],[11], they are known foremost for their roles in life span extension [10],[12]. Thus far, all of the known mutations and treatments that extend *C. elegans* reproductive period also regulate longevity [6],[7]. In addition, the link between longevity and reproduction has been suggested and/or reported in multiple organisms [37]–[43]. These data suggest the possibility that the regulation of life span and reproductive span are coupled, or even regulated by the same mechanisms. Our TGF-ß Dauer pathway data further support this notion, as the mutants increase both life span and reproductive span (Figure 4E) [19].

**Figure 5:**
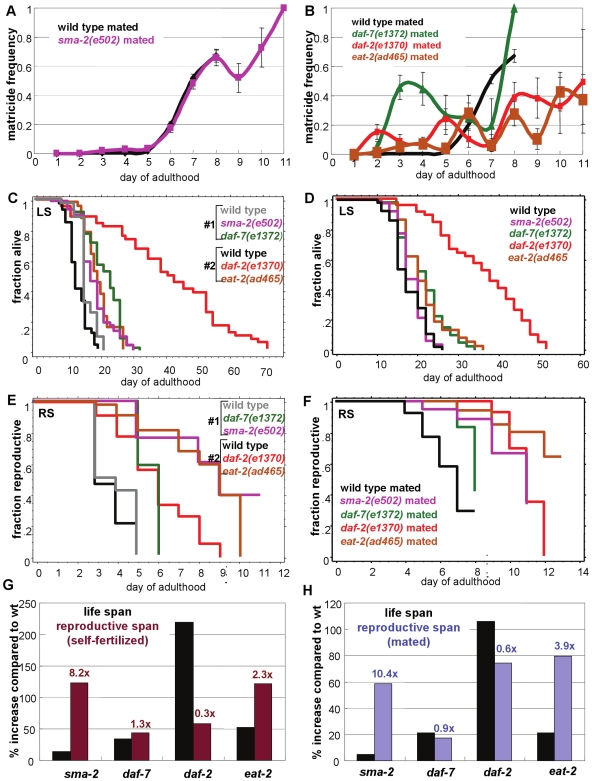
TGF-ß Sma/Mab mutants extend reproductive span most disproportionately to longevity effects. (A, B) Matricide frequencies with age of wild-type, *sma-2*, *daf-7*, *daf-2*, and *eat-2* animals (±SEP). Wild-type and *sma-2* have similar rates of increasing matricide frequency with age, while *daf-2* and *eat-2* rates are lower, and *daf-7's* Egl defect causes very early matricide. (C, D) The life span of *sma-2* is shorter than *daf-7*, *daf-2*, and *eat-2*. (E, F) The reproductive span of *sma-2* is either longer or comparable to these mutants when self-fertilized (E) or mated (F). (G, H) Percent change from wild-type life span and self-fertilized reproductive span (G) or mated reproductive span (H) of *sma-2*, *daf-7*, *daf-2*, and *eat-2*. Numbers above reproductive span bars indicate the fold of reproductive span increase over life span increase. *sma-2* has the largest effect on reproductive span, compared with its effect on life span. Note that (G) is calculated from (C, E), and (H) is calculated from (D, F). Additional statistics presented in Table S7 and Table S8.

However, we found that Sma/Mab pathway mutants only mildly affect longevity, despite their dramatic effects on reproductive span. Some Sma/Mab mutants increased life span moderately (*dbl-1*, *sma-6*) or inconsistently (*sma-2*, *sma-4*), while others appeared to have no effect on longevity (*sma-3*, *sma-9*) (Figure 4A–4D; Table S6, S7, S8, S9, S10). Because these alleles are not nulls, the inconsistencies in longevity between mutants in the pathway could be due to varying hypomorphic effects. Therefore we compared each mutant allele's effect on reproductive span and life span (Figure 4E). While *daf-4* mutants doubled both reproductive span and life span, and *daf-7* mutations had a moderate effect on each, exclusive members of the Sma/Mab pathway disproportionately extended reproductive span compared to life span (Figure 4E). This effect was maintained when the mated reproductive spans were considered (compare *sma-2* and *daf-7* in Figures 4E, Figure 5G and 5H). *daf-4's* effect on the two processes is likely due to its dual roles in TGF-ß Dauer regulation of life span [19] and in TGF-ß Sma/Mab regulation of reproductive span.

Matricide is a common event in the late reproductive period, but we noticed that the TGF-ß mutants are different from longevity mutants in this regard. In mated wild-type animals, the matricide frequency increased with age within the reproductive period (Figure 5A), perhaps reflecting aging of the musculature required for egg-laying. After day 9, reproduction stopped completely, therefore no matricide was observed. The matricide frequency of *sma-2* mutants also increased with age at a similar rate as wild type (Figure 5A). Because *sma-2* mutants continued to reproduce, however, the matricide rate continued to rise further, and therefore more mutant animals suffered from matricide than wild type. The matricide frequency of *daf-2* and *eat-2* mutants, however, increased at a much slower rate (Figure 5B). For example, on day 8 about 70% of worms died from matricide in both wild type and *sma-2* animals, whereas only 40% of *daf-2* and 30% of *eat-2* animals died of matricide (Figure 5A and 5B). (*daf-7* mutants exhibited high matricide rate from very early age (Figure 5B), due to their Egl defects (Figure 1D), therefore are different from the other strains.) The matricide frequency data suggest that late-reproduction matricide may be a somatically-controlled event, separate from reproductive aging. Together with the life span data, the matricide data suggest that *sma-2*'s soma ages at a rate that is similar to wild type, unlike the *daf-2* and *eat-2* longevity mutants.

To further investigate *sma-2*'s role in somatic aging, we compared the effects of mutations in *sma-2*, *daf-2*, *eat-2*, and *daf-7* on life span and reproductive span. The other mutants have longer life spans than *sma-2* (Figure 5C and 5D; Table S7 and Table S8), but their reproductive spans are either shorter or comparable to *sma-2* (Figure 5E and 5F; Table S7, S8). In fact, *daf-2* animals increase life span to a greater degree than reproductive span (Figure 5G and 5H), while *sma-2* and *eat-2* have greater effects on reproductive span than life span. *sma-2*'s effect on reproductive span is the most disproportionate among all the mutants. For example, when comparing the increases in mated reproductive spans and life spans of all the mutants (Figure 5H), *sma-2*'s increase in reproductive span is 10-fold its increase in lifespan, whereas *eat-2*'s effect on reproductive span is only 4-fold, and the *daf-7*'s and *daf-2*'s are both less than one fold. Together, our data show that TGF-ß Sma/Mab signaling affects reproductive aging disproportionately to its effect on longevity compared to other reproductive span and life span mutants.

### Sma/Mab signaling does not regulate reproductive aging through IIS or DR somatic aging pathways

**Figure 6:**
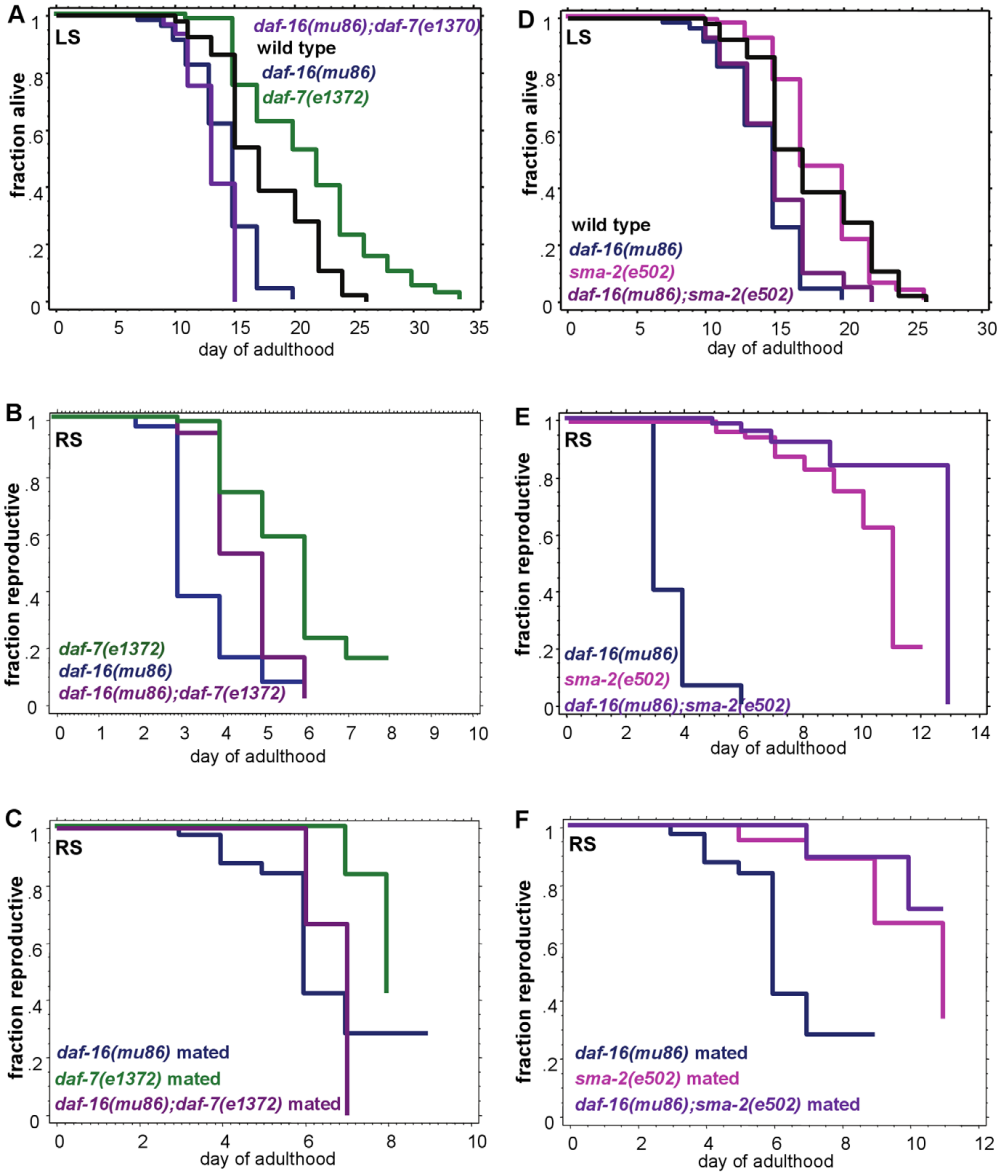
TGF-ß Sma/Mab signaling regulates reproductive span independently of DAF-16/FOXO activity. (A–C) *daf-7's* life span (A), self-fertilized reproductive span (B), and mated reproductive span (C) are significantly suppressed by loss of *daf-16* activity. (D–F) *sma-2*'s life span (D) is significantly suppressed by *daf-16* mutation, but its self-fertilized reproductive span (E) and mated reproductive span (F) are not suppressed by loss of *daf-16* activity. (Note that Figure 6A and 6D and Figure 5D are from one experiment; Figure 6C and 6F and Figure 5F are from one experiment.) Additional statistics in Table S9. Matricide data presented in Figure S7B.

The FOXO transcription factor DAF-16 is required for longevity of the IIS pathway mutant *daf-2* [10] and is also required for *daf-2's* effects on reproduction [7] (W. Shaw & C.T. Murphy, unpublished data). Previously, we showed that TGF-ß Dauer signaling regulates longevity through its interactions with the IIS pathway, as the lifespan extension of *daf-7* mutants is suppressed by loss of DAF-16/FOXO transcription factor activity [19] (Figure 6A). To test the role of *daf-16* in TGF-ß Dauer pathway regulation of reproduction, we compared the reproductive spans of *daf-7(e1372)*, *daf-16(mu86)*, and *daf-16(mu86);daf-7(e1372)* double mutants. We found that *daf-7's* reproductive span extension was significantly suppressed by loss of *daf-16* activity (Figure 6B and 6C; Table S9), suggesting that DAF-16/FOXO activity is required for both the life span and reproductive span extensions of *daf-7* mutants. Interestingly, loss of *daf-16* activity also suppressed *sma-2*'s small life span extension (Figure 6D, Figure S7A; Table S9); *sma-2's* occasional moderate effect on longevity may be due to cross-talk between the TGF-ß and IIS pathways rather than a primary output of TGF-ß Sma/Mab signaling, reminiscent of the cross-talk between the two TGF-ß pathways in dauer regulation [44],[45], and TGF-ß Dauer/IIS cross-talk in longevity regulation [19]. By contrast with its effect on *sma-2* life span, loss of *daf-16* activity failed to suppress *sma-2* and *dbl-1* self-fertilized and mated reproductive span extensions (Figure 6E and 6F; Figure S7C and S7D; Table S9). However, the double mutant's peak matricide frequency shifted earlier (Figure S7B), consistent with *daf-16;sma-2* and *daf-16's* shorter life spans. These results suggest that the TGF-ß Sma/Mab pathway's regulation of reproductive span is not mediated by DAF-16/FOXO activity. Together with its disproportionate effect on the two processes, the TGF-ß Sma/Mab pathway appears to have genetically uncoupled regulation of reproduction and longevity.

**Figure 7:**
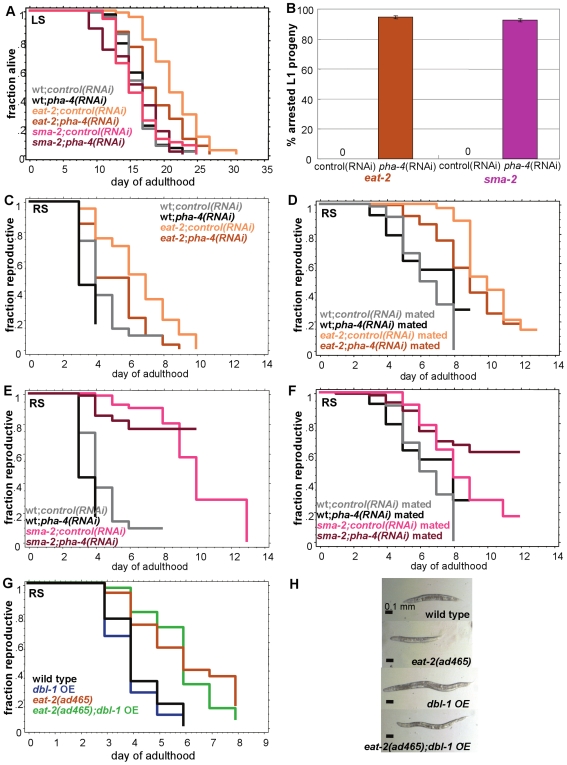
TGF-ß Sma/Mab pathway regulates reproductive span independently of dietary restriction. (A) *pha-4* RNAi suppresses *eat-2(ad465)*'s life span, but has no effect on *sma-2(e502)*'s life span. (B) *sma-2(e502)* animals produce similar percentage of arrested L1 progeny as *eat-2(ad465)* animals when treated with *pha-4* RNAi (±SEP, p = 0.17, n = 177 for *sma-2*, n = 182 for *eat-2*). When treated with control RNAi, no arrested L1s are produced. (C, D) *eat-2(ad465)*'s self-fertilized reproductive span (C) and mated reproductive span (D) are both significantly suppressed by *pha-4* RNAi treatment. (E, F) Neither *sma-2(e502)*'s self-fertilized reproductive span (E) nor mated reproductive span (F) are suppressed by loss of *pha-4* activity. (Note that (C, E) are from one experiment, while (D, F) are from another.) Additional statistics presented in Table S10. (G) *dbl-1* over-expression does not suppress *eat-2(ad465)*'s reproductive span extension. Additional statistics are in Table S11. (H) Body size of wild type, *eat-2(ad465)*, *dbl-1 OE*, and *eat-2(ad465);dbl-1 OE*. *eat-2;dbl-1 OE* animals are larger than wild type. *sma-2;pha-4(RNAi)* animals have a severe egg-laying defect, possibly masking even longer reproductive spans (Figure S8).

The FoxA transcription factor PHA-4 is required for the life span extension of the Dietary Restriction model *eat-2* (Figure 7A) [46]. We found that *eat-2's* reproductive span extension was also significantly suppressed by loss of PHA-4 activity (Figure 7C and 7D; Table S10) when treated from L4 onward. (To check the efficacy of *pha-4* RNAi, we determined the fraction of arrested L1 progeny from L4-onward-fed mothers, and found that *sma-2* and *eat-2* animals are similarly sensitive to *pha-4* RNAi (Figure 7B).) To test whether TGF-ß Sma/Mab signaling utilizes the Dietary Restriction pathway, we tested *sma-2's* requirement for PHA-4 activity. In contrast to *eat-2's* requirement for *pha-4*, we found that *pha-4* RNAi treatment did not suppress the reproductive span extension of *sma-2* mutants (Figure 7E and 7F; Table S10). Thus, while *pha-4* is required for the reproductive span changes associated with Dietary Restriction, it is not required for *sma-2*'s reproductive span extension.

If the reproductive span extension of Dietary Restriction animals required TGF-ß Sma/Mab signaling, we might expect the DBL-1 ligand to act downstream of the *eat-2*-induced DR effect, which is caused by *eat-2* mutants' inability to pump their pharynx to properly ingest food. However, we observed that over-expression of the DBL-1 ligand is not sufficient to suppress *eat-2*'s reproductive span extension (Figure 7G; Table S11), consistent with the hypothesis that Dietary Restriction and TGF-ß Sma/Mab regulate reproductive span independently. (Interestingly, the *eat-2;dbl-1 OE* animals, like *dbl-1 OE* single mutants, are larger than wild type (Figure 7H) yet have a long reproductive span, further supporting the notion that there is no direct correlation between body size and reproductive span.)

Our results show that in addition to its role in body size regulation, the TGF-ß Sma/Mab pathway is a novel regulator of reproductive aging. Sma/Mab signaling regulates reproductive aging independently of at least two known regulators of somatic aging, Dietary Restriction and IIS/FOXO signaling, and uncouples reproductive aging from somatic aging.

## Discussion

### TGF-ß Sma/Mab signaling is a novel regulator of reproductive aging

Here we have shown that loss of function of a canonical TGF-ß pathway significantly delays *C. elegans* reproductive aging. Intriguingly, TGF-ß Sma/Mab mutants extend reproductive span without a proportional extension of life span. We have also found that TGF-ß Sma/Mab reproductive span extension is genetically independent of regulation by Dietary Restriction and Insulin/IGF-1 Signaling. The uncoupling of reproductive and somatic aging in TGF-ß Sma/Mab signaling mutants suggests that the molecular mechanisms underlying the maintenance of somatic [47],[48] and reproductive tissues are distinct. This is the first identification of a pathway that regulates reproductive aging independently of somatic aging, and may lead to insights into mechanisms that specifically govern age-related reproductive cessation.

### Somatic aging and reproductive aging may be coupled

Signaling from the reproductive system to the soma to regulate aging has already been shown through germ line and somatic gonad ablation experiments in worms and flies [49],[50]. Because germ line and somatic gonad ablation both result in sterility, but have opposite effects on life span, direct resource allocation from the germ line to the soma cannot be the cause of this longevity. Instead, signals from the germ line and somatic gonad normally communicate with the rest of the soma to regulate life span, acting through the insulin/FOXO and *daf-12* nuclear hormone pathways [49]–[51]. Our results suggest that the reproductive system may also normally receive signals through these regulatory pathways and the TGF-ß Sma/Mab pathway to regulate its rate of aging, allowing the animal to adjust its reproductive rate to its environment.

The prevalence of matricide by the TGF-ß Sma/Mab mutants illustrates the importance of signaling that normally links reproductive and somatic aging. Many animals slow their reproductive rates in response to environmental factors, such as high predation and food shortages, in order to optimize their reproductive fitness [7],[16],[18],[52]. In some cases, such as under environmental stress, late reproduction can increase fitness, allowing increased genetic diversity through mating (“facultative outcrossing”) [17]. Like *daf-2* and *eat-2* mutants, reduced TGF-ß Sma/Mab signaling slows the rate of reproductive aging and thus extends reproductive span. However, TGF-ß Sma/Mab mutants do not concomitantly slow somatic aging, and as a result they often suffer from high reproductive-age mortality induced by the physical stresses of reproduction, whereas same-aged longevity mutants *daf-2* and *eat-2* experience less age-related matricide (see Figures 2D and 2E and Figure 5A and 5B). From these results we infer that the slowing of somatic aging is required for successful delayed reproduction, which may be necessary under certain environmental conditions. Signals through the TGF-ß Sma/Mab pathway may allow the animal to adjust its reproductive rate to its environment. In turn, signals from the germ line and somatic gonad coordinate somatic aging rate with reproductive aging rate to allow successful reproduction.

### Worms and humans utilize similar mechanisms to regulate post-reproductive life span

If reproductive and somatic aging are coupled, why do worms and humans live so long after reproduction has ceased? A popular but controversial theory specific to humans postulates that investment in grand-progeny by grandmothers increases fitness to a greater degree than would continuing reproduction, and thus downregulation of reproductive ability in mid-life is evolutionarily beneficial (the “Grandmother Hypothesis” [53],[54]). However, as *C. elegans* does not care for its young, such an investment in its grand-progeny cannot explain its similarly early decline in reproduction and long post-reproductive life span.

Instead, we propose that reproduction itself requires the soma to function at its highest level, but the body can survive well below this threshold level of function. That is, if one were to plot every parameter of function (reproduction, motility, pathogen resistance, survival, etc.) in both worms and humans, these functions would all peak during the reproductive period, but begin to decline post-reproductively, each at a different rate. Successful reproduction requires peak physical condition of the soma; the increased matricide rates in older, reproductive Sma/Mab mutants shows that increased oocyte quality in the absence of healthy somatic tissues can be catastrophic, at least to the mother and any unproduced late progeny. By contrast, “survival” is the lowest measurable function (assayed as live vs. dead) and thus persists much longer than reproductive activity. Humans survive well past the age of peak physical function, a period that overlaps with female reproduction. Improvements in medicine, nutrition, hygiene, and environment have extended human life span significantly [55],[56], extending the post-reproductive life span but having little effect on maximum reproductive span. Analogously, the worm's life span in the low-predation and low-pathogen conditions of the laboratory is likely longer than in the wild, but likely largely affects post-reproductive life span. Therefore, the long post-reproductive life span of both worms and human females could be attributed to the high level of somatic function required for successful reproduction, essentially a side effect of the requirements for successful reproduction earlier in life.

### Regulation of reproductive aging may be evolutionarily conserved

Longevity is regulated by insulin/IGF-1/FOXO signaling and by Dietary Restriction in worms through mammals [10], [12], [57]–[59], despite large differences in chronological life spans of these organisms. Additionally, Insulin/IGF-1 Signaling and Dietary Restriction have been implicated in regulation of mammalian reproductive aging [14],[52],[60],[61]. Intriguingly, TGF-ß levels are upregulated in aged mouse oocytes [62], and TGF-ß activity regulates mammalian follicle cell activity [63]. Thus, it is possible that regulation of reproductive aging, like the regulation of somatic aging by IIS and DR pathways, is evolutionarily conserved, and that TGF-ß signaling may regulate human reproductive cessation.

Despite the vast differences in their life histories and chronological time frames, our work suggests that the regulation of worms and humans' longevity and reproductive spans may be conserved. Future studies will determine whether Sma/Mab mutants use a conserved mechanism to slow reproductive aging in *C. elegans*. If so, modulation of TGF-ß signaling may offer new avenues to improve fertility and offspring health in mothers of advanced age.

## Materials and Methods

### *C. elegans* genetics

All strains were cultured using standard methods [64]. In all experiments, N2 is the wild type. LG I: *daf-16(mu86)*, *daf-8(e1393)*. LG II: *eat-2(ad465)*, *rrf-3(pk1426)*, *sma-6(wk7)*. LG III: *daf-2(e1370)*, *daf-7(e1372)*, *daf-4(e1364)*, *sma-2(e502)*, *sma-3(wk28)*, *sma-3(wk20)*, *sma-4(e729)*. LG IV: *daf-1(m40)*, *daf-14(m77)*, *fem-1(hc17)*. LG V: *dbl-1(nk3)*, *dbl-1(wk70)*. LG X: *sma-9(qc3)*, *sma-9(wk55)*.

Strains: BW1940: *ctIs40 X [ZC421(+) containing dbl-1;sur-5::gfp]*. CQ33: *eat-2(ad465) II*; *ctIs40 X [ZC421(+) containing dbl-1;sur-5::gfp]*. CQ17: *daf-1(m40) IV* outcrossed to N2 3×. CQ16: *daf-7(e1372) III* outcrossed to N2 3×. CQ14: *daf*-*14*(*m77*) *IV* outcrossed to N2 3×. CQ19: *sma-2(e502) III* outcrossed to N2 3×. CQ18: *sma-9(wk55) X* outcrossed to N2 3×. CQ53: *sma-2(e502);fem-1(hc17)*.

CQ49: *daf-16(mu86);sma-2(e502)*. CQ25: *daf-16(mu86);daf-7(e1372)*. CF1041: *daf-2(e1370) III*.

### Reproductive span analysis

Individual synchronized L4 hermaphrodites were moved to fresh plates daily until reproduction ceased for at least two days. The last day of viable progeny production was noted as the day of reproduction cessation for each individual. When matricide occurred, the animal was censored from the experiment on that day. All experiments were performed at 20°C, except that *sma-2(e502);fem-1(hc17)* and *fem-1(hc17)* worms were shifted to 25°C from L3 and back to 20°C after L4. All experiments were performed with at least 10 individuals per strain (most experiments included >25 individuals, as indicated in Supplementary Tables). The log-rank (Mantel-Cox) method was used to test the null hypothesis. In mating reproductive span assays, L4 hermaphrodites were mated to young wild-type males at a 1∶3 ratio for 24 hours before being separated onto individual plates. Successful mating was ascertained by the fraction of male progeny each day. For the *pha-4* RNAi reproductive span experiments, mothers were moved onto RNAi bacteria starting at L4.

### Progeny production analysis

Individual synchronized L4 hermaphrodites were moved to fresh plates and the number of progeny produced by each individual was counted daily until reproduction ceased for at least two days. When matricide occurred, the animal was censored from the experiment on that day. All experiments were performed at 20°C with at least 6 individuals per strain (most experiments included 20–40 individuals).

### Matricide (bagging) rate

The assay was performed as described in Reproductive Span analysis, except the cumulative percentage of hermaphrodites that underwent matricide was calculated daily. The matricide frequency was determined as the frequency of reproductive worms that die of matricide; as matricide is caused by internal progeny hatching, non-reproductive worms by definition never die of matricide, and thus are not included in calculation. This number reflects the likelihood of matricide.

### Hatching rate

Eggs were synchronized by hypochlorite treatment and allowed to develop at 20°C until day 3 of adulthood. ∼100 synchronized hermaphrodites were transferred to a new plate and allowed to lay progeny for 1 hour; eggs and L1 progeny were counted at 3-hour intervals.

### Survival analysis

The first day of adulthood was defined as t = 0, and the log-rank (Mantel-Cox) method was used to test the null hypothesis in Kaplan-Meier survival analysis, as previously described [65]. All experiments were carried out at 20°C with 50 µM FUdR starting at L4; n>60 in each experiment.

### RNA interference

Bacterial feeding RNAi experiments were carried out as previously described [66] with IPTG at 1 mM. Each clone was verified by PCR and sequence analysis.

### *pha-4* RNAi efficacy

*pha-4* RNAi efficacy was determined by counting the arrested L1s produced by mothers fed from L4 onward compared with control vector.

## Supporting Information

Figure S1 TGF-β Dauer pathway mutants have only moderate effects on reproductive span. (A) Schematic representation of the TGF-β Dauer pathway in *C. elegans*, with mouse homologs in parentheses. (B, C) Self-fertilized reproductive spans of TGF-β Dauer pathway mutants. (D) Percent increase in reproductive span of TGF-β Dauer pathway mutants over wild type. (E–G) Progeny production profiles of TGF-β Dauer pathway mutants. Statistics in Table S2.
(1.56 MB TIF)

Figure S2 TGF-β Sma/Mab pathway regulates reproductive span. (A) Schematic representation of the TGF-β Sma/Mab pathway in *C. elegans*, with mouse homologs in parentheses. (B, C) Additional self-fertilized reproductive spans of the TGF-β Sma/Mab pathway mutants. (D) *sma-2* and *dbl-1* animals mated with wild-type males at day 4 still significantly extend reproductive span (p<0.0001 for each). (E) A higher percentage of Sma/Mab pathway mutants die of matricide than wild type.
(1.56 MB TIF)

Figure S3 Progeny production profiles of TGF-β Sma/Mab pathway mutants. Self-fertilized animals (A–C) and wild-type mated animals (D, E).
(1.56 MB TIF)

Figure S4 TGF-β Sma/Mab mutants display small body size, slow ovulation, and reduced progeny number. (A) Example images comparing body size of wild type and three TGF-β Sma/Mab mutants. Scale bar represents 1 mm. (B) Average body length of genotypes in (A); mean±SEM. (C) Mated *sma-2* animals ovulate slower than wild type. (D–F) Mated *sma-2*, *dbl-1*, and *sma-3* mutations reduce progeny production.
(1.56 MB TIF)

Figure S5 TGF-β Sma/Mab pathway regulates late reproduction in adulthood. (A) Progeny production profile of *sma-9* (Ahringer clone) RNAi whole-life and adult-only treated animals in late reproductive life. (B) Progeny number produced in late reproductive life, data from (A). (C) Last day of reproduction in *sma-9* (Ahringer clone) RNAi whole-life and adult-only treated wild type or rrf-3 animals.
(1.56 MB TIF)

Figure S6 Five non-TGF-β small mutants. (A) Example images comparing body size of wild type and five other non-TGF-β small mutants. Scale bar represents 1 mm. (B) Average body length of genotypes in (A); mean±SEM.
(1.56 MB TIF)

Figure S7 TGF-β Sma/Mab pathway mutants extend reproductive span independently of DAF-16/FOXO activity. (A) *daf-16(mu86)* mutation suppresses *sma-2's* life span. (B) *daf-16* and *daf-16;sma-2* mutants increase matricide frequencies earlier. (C) *daf-16* RNAi does not suppress *sma-2's* reproductive span. (D) *daf-16* RNAi does not suppress *dbl-1's* reproductive span. Statistics in Table S9.
(1.56 MB TIF)

Figure S8 *sma-2;pha-4(RNAi)* animals' body cavity are filled with unhatched eggs (white arrows). *sma-2;pha-4(RNAi)* animals have a severe egg-laying defect not observed in *eat-2* or wild-type worms treated with *pha-4* RNAi; many hermaphrodites were censored from the assay when their body cavity filled with arrested eggs, causing maternal death. This defect may have masked longer reproductive spans of the *sma-2(e502);pha-4(RNAi)* animals.
(1.56 MB TIF)

Table S1 TGF-β Sma/Mab pathway self-fertilized and wild-type mated reproductive spans.
(0.10 MB PDF)

Table S2 Self-fertilized reproductive spans (RS) of TGF-β Dauer pathway mutants.
(0.09 MB PDF)

Table S3 Self-fertilized brood size of TGF-β Sma/Mab pathway mutants.
(0.09 MB PDF)

Table S4 Mated brood size of TGF-β Sma/Mab pathway mutants.
(0.09 MB PDF)

Table S5 Mated reproductive spans (RS) of non-TGF-β small mutants.
(0.07 MB PDF)

Table S6 Life spans (LS) of TGF-β Sma/Mab pathway mutants.
(0.09 MB PDF)

Table S7 Comparison of life spans and self-fertilized reproductive spans in mutants with extended reproductive spans.
(0.10 MB PDF)

Table S8 Comparison of life spans and wild-type mated reproductive spans in mutants with extended reproductive spans.
(0.08 MB PDF)

Table S9 Effects of *daf-16* loss-of-function on life spans and reproductive spans in TGF-β Dauer and Sma/Mab mutants.
(0.10 MB PDF)

Table S10 Effects of *pha-4* loss-of-function on life spans and reproductive spans in *eat-2* and TGF-β Sma/Mab mutants.
(0.10 MB PDF)

Table S11 Effects of *dbl-1* over-expression on the reproductive span of *eat-2* mutants.
(0.07 MB PDF)
