# Supplementary figures and images for: TGF-ß Sma/Mab Signaling Mutations Uncouple Reproductive Aging from Somatic Aging
Source: PLoS Genet. 2009 Dec 24;5(12):e1000789. doi: 10.1371/journal.pgen.1000789 (PMC2791159; doi:10.1371/journal.pgen.1000789)

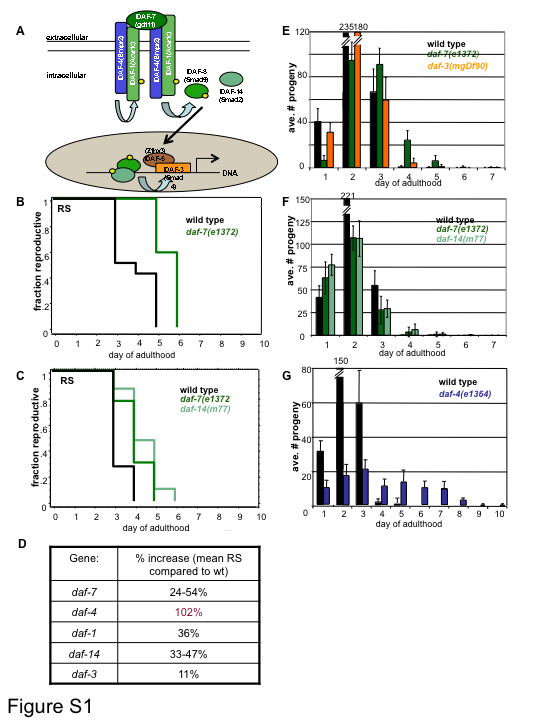

Supplement: Figure S1 — TGF-β Dauer pathway mutants have only moderate effects on reproductive span. (A) Schematic representation of the TGF-β Dauer pathway in C. elegans, with mouse homologs in parentheses. (B, C) Self-fertilized reproductive spans of TGF-β Dauer pathway mutants. (D) Percent increase in reproductive span of TGF-β Dauer pathway mutants over wild type. (E–G) Progeny production profiles of TGF-β Dauer pathway mutants. Statistics in Table S2. (1.56 MB TIF) [file pgen.1000789.s001.tif]

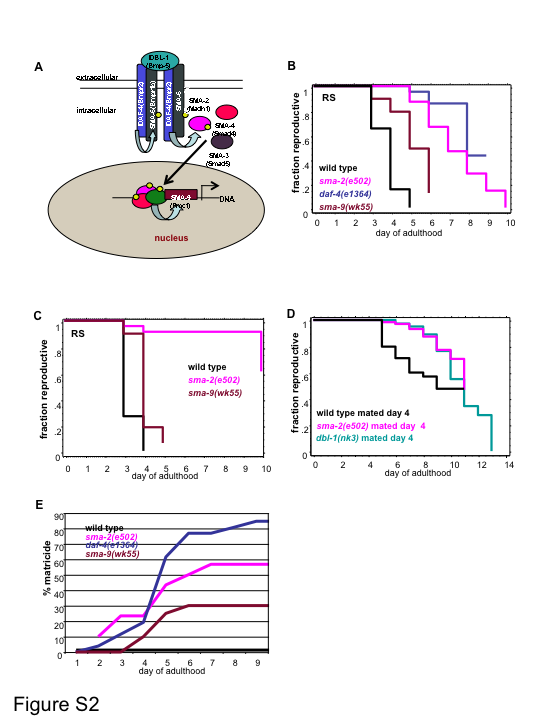

Supplement: Figure S2 — TGF-β Sma/Mab pathway regulates reproductive span. (A) Schematic representation of the TGF-β Sma/Mab pathway in C. elegans, with mouse homologs in parentheses. (B, C) Additional self-fertilized reproductive spans of the TGF-β Sma/Mab pathway mutants. (D) sma-2 and dbl-1 animals mated with wild-type males at day 4 still significantly extend reproductive span (p<0.0001 for each). (E) A higher percentage of Sma/Mab pathway mutants die of matricide than wild type. (1.56 MB TIF) [file pgen.1000789.s002.tif]

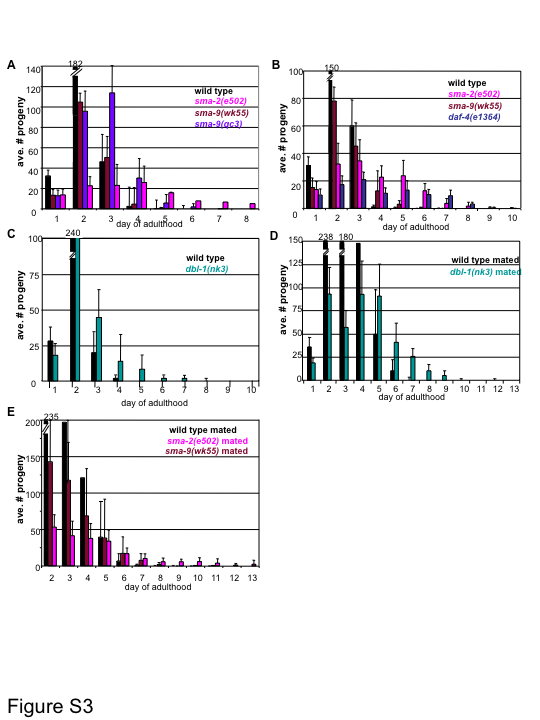

Supplement: Figure S3 — Progeny production profiles of TGF-β Sma/Mab pathway mutants. Self-fertilized animals (A–C) and wild-type mated animals (D, E). (1.56 MB TIF) [file pgen.1000789.s003.tif]

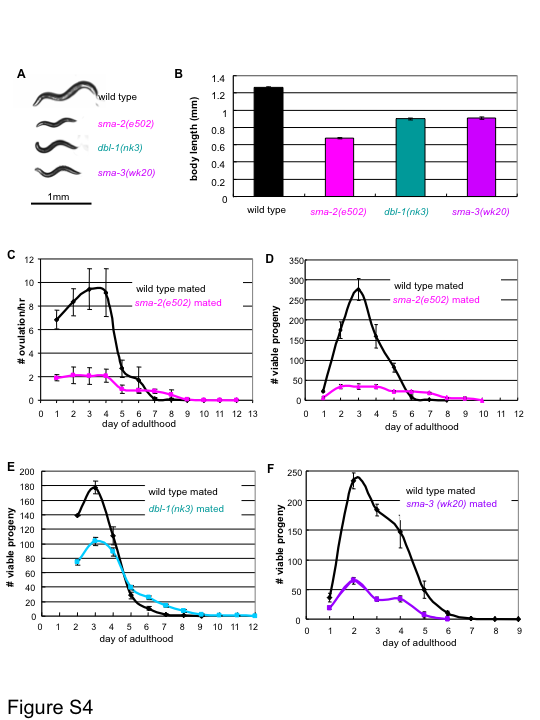

Supplement: Figure S4 — TGF-β Sma/Mab mutants display small body size, slow ovulation, and reduced progeny number. (A) Example images comparing body size of wild type and three TGF-β Sma/Mab mutants. Scale bar represents 1 mm. (B) Average body length of genotypes in (A); mean±SEM. (C) Mated sma-2 animals ovulate slower than wild type. (D–F) Mated sma-2, dbl-1, and sma-3 mutations reduce progeny production. (1.56 MB TIF) [file pgen.1000789.s004.tif]

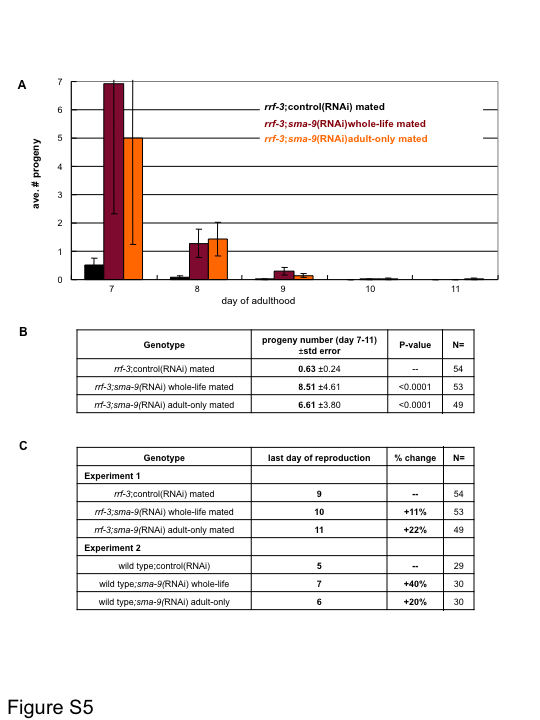

Supplement: Figure S5 — TGF-β Sma/Mab pathway regulates late reproduction in adulthood. (A) Progeny production profile of sma-9 (Ahringer clone) RNAi whole-life and adult-only treated animals in late reproductive life. (B) Progeny number produced in late reproductive life, data from (A). (C) Last day of reproduction in sma-9 (Ahringer clone) RNAi whole-life and adult-only treated wild type or rrf-3 animals. (1.56 MB TIF) [file pgen.1000789.s005.tif]

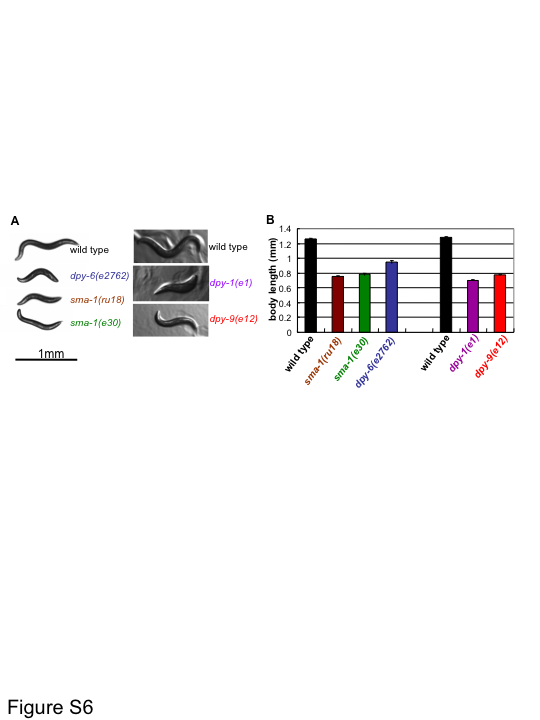

Supplement: Figure S6 — Five non-TGF-β small mutants. (A) Example images comparing body size of wild type and five other non-TGF-β small mutants. Scale bar represents 1 mm. (B) Average body length of genotypes in (A); mean±SEM. (1.56 MB TIF) [file pgen.1000789.s006.tif]

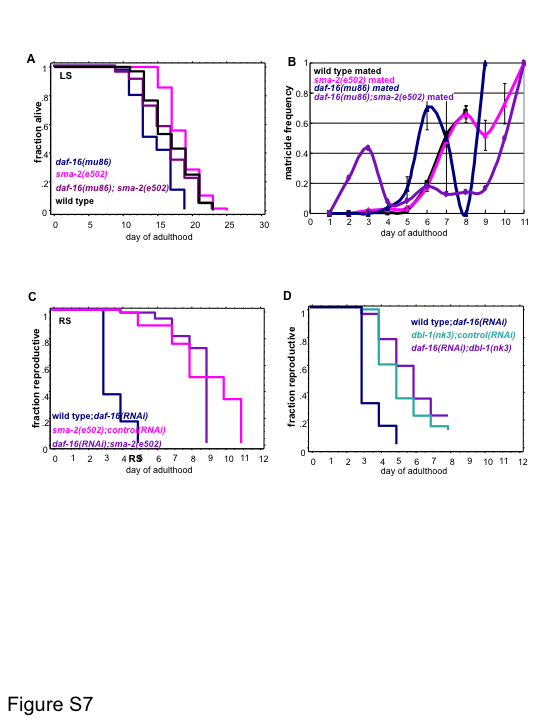

Supplement: Figure S7 — TGF-β Sma/Mab pathway mutants extend reproductive span independently of DAF-16/FOXO activity. (A) daf-16(mu86) mutation suppresses sma-2's life span. (B) daf-16 and daf-16;sma-2 mutants increase matricide frequencies earlier. (C) daf-16 RNAi does not suppress sma-2's reproductive span. (D) daf-16 RNAi does not suppress dbl-1's reproductive span. Statistics in Table S9. (1.56 MB TIF) [file pgen.1000789.s007.tif]

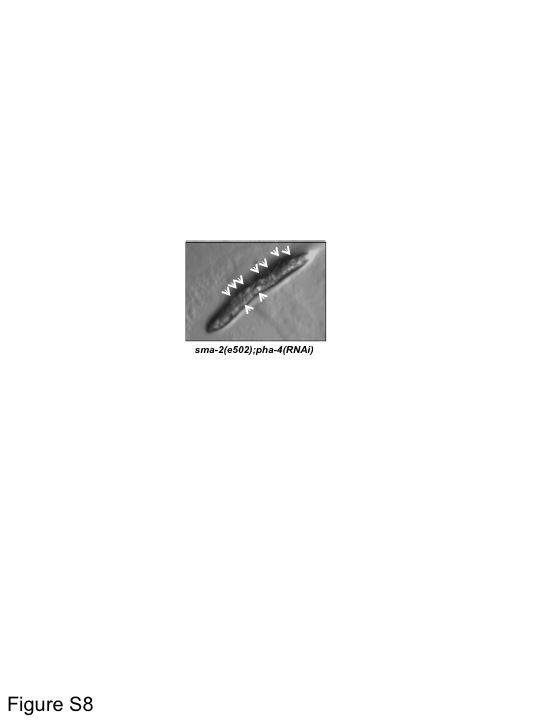

Supplement: Figure S8 — sma-2;pha-4(RNAi) animals' body cavity are filled with unhatched eggs (white arrows). sma-2;pha-4(RNAi) animals have a severe egg-laying defect not observed in eat-2 or wild-type worms treated with pha-4 RNAi; many hermaphrodites were censored from the assay when their body cavity filled with arrested eggs, causing maternal death. This defect may have masked longer reproductive spans of the sma-2(e502);pha-4(RNAi) animals. (1.56 MB TIF) [file pgen.1000789.s008.tif]
