# Supplementary material for: TGF-ß Sma/Mab Signaling Mutations Uncouple Reproductive Aging from Somatic Aging
Source: PLoS Genet. 2009 Dec 24;5(12):e1000789. doi: 10.1371/journal.pgen.1000789 (PMC2791159; doi:10.1371/journal.pgen.1000789)
Supplement: Table S1 — TGF-β Sma/Mab pathway self-fertilized and wild-type mated reproductive spans. (0.10 MB PDF) [file pgen.1000789.s009.pdf]

Genotype	mean RS± std error	% change	P-value	N=
**Experiment 1:**				
wild type	**3.6** ±0.2	**--**	--	30
sma-4(e729)	*	*	ND	25
sma-2(e502)	**7.4** ±0.2	**+106%**	<0.0001	26
**Experiment 2:**				
wild type	**3.8** ±0.2	**--**	--	33
dbl-1(nk3)	**7.2**±0.3	**+89%**	<0.0001	33
sma-3(wk20)	**5.9** ±0.3	**+55%**	0.0042	48
sma-3(wk28)	*	*	ND	15
**Experiment 3:**				
wild type	**3.8** ±0.2	**--**	--	45
daf-4(e1364)	**7.7** ±0.3	**+103%**	<0.0001	26
sma-2(e502)	**7.4** ±0.4	**+95%**	<0.0001	30
sma-9(wk55)	**5.2** ±0.2	**+37%**	<0.0001	19
**Experiment 4:**				
wild type	**3.3** ±0.1	**--**	--	30
sma-2(e502)	**9.4** ±0.5	**+185%**	<0.0001	26
sma-9(wk55)	**4.1** ±0.1	**+24%**	<0.0001	30
**Experiment 5 (mated):**				
wild type	**6.0** ±0.4	**--**	--	29
sma-2(e502)	**11.4** ±0.6	**+90%**	<0.0001	28
**Experiment 6 (mated):**				
wild type	**5.6** ±0.5	**--**	--	15
sma-2(e502)	**10.1** ±0.8	**+80%**	0.0004	11
sma-9(wk55)	**6.7** ±0.6	**+20%**	0.06	14
**Experiment 7 (mated):**				
wild type	**6.1** ±0.2	**--**	--	40
dbl-1(nk3)	**10.2** ±0.3	**+67%**	<0.0001	71
**Experiment 8 (mated):**				
wild type	**6.0** ±0.3	**--**	--	15
dbl-1(nk3)	**11.6** ±0.3	**+93%**	<0.0001	67
sma-3(wk20)	*	*	0.0042	39
**Experiment 9 (mated):**				
fem-1(hc17)	**6.3** ±0.3	**--**	--	60
sma-2(e502);fem-1(hc17)	*	*	<0.0001	98

*Animals exhibit high matricide, obscuring ability to measure true RS (reproductive span).
