# Supplementary material for: TGF-ß Sma/Mab Signaling Mutations Uncouple Reproductive Aging from Somatic Aging
Source: PLoS Genet. 2009 Dec 24;5(12):e1000789. doi: 10.1371/journal.pgen.1000789 (PMC2791159; doi:10.1371/journal.pgen.1000789)
Supplement: Table S2 — Self-fertilized reproductive spans (RS) of TGF-β Dauer pathway mutants. (0.09 MB PDF) [file pgen.1000789.s010.pdf]

Genotype	mean RS± std. error	% change	P-value	N=
**Experiment 1:**				
wild type	**3.5**±0.2	**--**	--	30
daf-7(e1372)	**5.4**±0.1	**+54%**	<0.0001	30
**Experiment 2:**				
wild type	**3.6**±0.2	**--**	--	30
daf-1(m40)	**4.9**±0.1	**+36%**	<0.0001	30
daf-14(m77)	**5.3**±0.2	**+47%**	<0.0001	30
**Experiment 3:**				
wild type	**3.8** ±0.2	**--**	--	45
daf-4(e1364)	**7.7** ±0.3	**+103%**	<0.0001	26
**Experiment 4:**				
wild type	**3.9**±0.3	**--**	--	12
daf-7(e1372)	**5.6**±0.2	**+44%**	<0.0001	17
**Experiment 5:**				
wild type	**3.3**±0.1	**--**	--	30
daf-7(e1372)	**4.1**±0.2	**+24%**	<0.0001	30
daf-14(m77)	**4.4**±0.2	**+33%**	<0.0001	30
