# Supplementary material for: TGF-ß Sma/Mab Signaling Mutations Uncouple Reproductive Aging from Somatic Aging
Source: PLoS Genet. 2009 Dec 24;5(12):e1000789. doi: 10.1371/journal.pgen.1000789 (PMC2791159; doi:10.1371/journal.pgen.1000789)
Supplement: Table S3 — Self-fertilized brood size of TGF-β Sma/Mab pathway mutants. (0.09 MB PDF) [file pgen.1000789.s011.pdf]

Genotype	Ave # progeny ± std error	% change	N=
**Experiment 1:**			
wild type	**233** ±62	**--**	8
sma-2(e502)	**67** ±57	**-71%**	8
sma-9(qc3)	**156** ±34	**-33%**	8
sma-9(wk55)	**99** ±61	**-58%**	8
daf-4(e1364)	**33** ±14	**-86%**	8
**Experiment 2:**			
wild type	**263** ±19	**--**	10
sma-2(e502)	**77** ±55	**-71%**	10
sma-9(qc3)	**248** ±34	**-6%**	10
sma-9(wk55)	**162** ±44	**-38%**	10
**Experiment 3:**			
wild type	**243** ±62	**--**	20
sma-2(e502)	**122** ±65	**-50%**	30
sma-9(qc3)	**200**±38	**-18%**	26
sma-9(wk55)	**153**±34	**-37%**	20
daf-4(e1364)	**74**±31	**-70%**	26
**Experiment 4:**			
wild type	**326** ±37	**--**	30
sma-2(e502)	**114** ±36	**-65%**	30
sma-9(wk55)	**218** ±33	**-33%**	30
**Experiment 4**:			
wild type	**243** ±21	**--**	20
daf-4(e1364)	**74** ±31	**-70%**	26
