# Supplementary material for: TGF-ß Sma/Mab Signaling Mutations Uncouple Reproductive Aging from Somatic Aging
Source: PLoS Genet. 2009 Dec 24;5(12):e1000789. doi: 10.1371/journal.pgen.1000789 (PMC2791159; doi:10.1371/journal.pgen.1000789)
Supplement: Table S4 — Mated brood size of TGF-β Sma/Mab pathway mutants. (0.09 MB PDF) [file pgen.1000789.s012.pdf]

Genotype	Ave # progeny ± std error	% change	N=
**Experiment 1:**			
wild type mated	**504** ±137	**--**	13
dbl-1(nk3) mated	**352** ±97	**-30%**	14
**Experiment 2:**			
wild type mated	**662** ±127	**--**	11
dbl-1(nk3) mated	**434** ±67	**-34%**	16
*sma-3(wk20)*mated	**159** ±42	**-76%**	4
**Experiment 3:**			
wild type mated	**723** ±155	**--**	8
sma-2(e502) mated	**189** ±33	**-74%**	9
**Experiment 4:**			
wild type mated	**636** ±165	**--**	15
sma-2(e502) mated	**243** ±53	**-62%**	8
sma-9(wk55) mated	**399** ±116	**-37%**	12
