# Supplementary material for: TGF-ß Sma/Mab Signaling Mutations Uncouple Reproductive Aging from Somatic Aging
Source: PLoS Genet. 2009 Dec 24;5(12):e1000789. doi: 10.1371/journal.pgen.1000789 (PMC2791159; doi:10.1371/journal.pgen.1000789)
Supplement: Table S5 — Mated reproductive spans (RS) of non-TGF-β small mutants. (0.07 MB PDF) [file pgen.1000789.s013.pdf]

Genotype	mean RS± std error	% change	P-value	N=
**Experiment 1**				
wild type mated	**8.7** ±0.2	**--**	--	73
dpy-6(e2762) mated	**7.1** ±0.2	**-18%**	<0.0001	79
sma-1(ru18) mated	**7.5** ±0.2	**-14%**	0.0006	77
sma-1(e30) mated	**6.5** ±0.2	**-25%**	0.0506	68
**Experiment 2**				
wild type mated	**9.1** ±0.2	**--**		69
dpy-1(e1) mated	**7.4** ±0.3	**-19%**	0.0028	70
dpy-9(e12) mated	**5.1** ±0.2	**-44%**	<0.0001	61
