# Supplementary material for: TGF-ß Sma/Mab Signaling Mutations Uncouple Reproductive Aging from Somatic Aging
Source: PLoS Genet. 2009 Dec 24;5(12):e1000789. doi: 10.1371/journal.pgen.1000789 (PMC2791159; doi:10.1371/journal.pgen.1000789)
Supplement: Table S6 — Life spans (LS) of TGF-β Sma/Mab pathway mutants. (0.09 MB PDF) [file pgen.1000789.s014.pdf]

Genotype	mean LS± std. error	% change	P-value	N
**Experiment 1:**				
wild type	**15.7** ±0.3	**--**	--	72
sma-2(e502)	**16.5** ±0.6	**+5%**	0.095	72
sma-9(wk55)	**15.2** ±0.3	**-3%**	0.22	71
sma-9(qc3)	**15.5** ±0.4	**-1%**	0.75	69
**Experiment 2:**				
wild type	**16.8** ±0.4	**--**	--	88
dbl-1(nk3)	**20.5** ±0.5	**+22%**	<0.0001	82
dbl-1(wk70)	**17.5** ±0.4	**+4%**	0.16	79
dbl-1 OE	**13.6** ±0.4	**-19%**	<0.0001	64
sma-6(wk7)	**20.0** ±0.3	**+19%**	<0.0001	80
sma-2(e502)	**22.3** ±0.5	**+33%**	<0.0001	84
sma-3(wk28)	**15 .4**±0.6	**-8%**	0.089	65
sma-3(wk20)	**17.8** ±0.5	**+6%**	0.045	76
sma-4(e729)	**23.0** ±0.5	**+37%**	<0.0001	77
sma-9(qc3)	**16.4** ±0.3	**-2%**	0.008	85
sma-9(wk55)	**15.6** ±0.3	**-7%**	0.13	80
**Experiment 3:**				
wild type	**13.5** ±0.3	**--**	--	75
sma-4(e729)	**13.8** ±0.8	**+2%**	0.74	70
**Experiment 4:**				
wild type	**14.4** ±0.3	**--**	--	73
dbl-1(nk3)	**20.0**±0.9	**+39%**	<0.0001	70
dbl-1 OE	**13.3** ±0.6	**-8%**	0.027	73
sma-6(wk7)	**15.7** ±0.5	**+9%**	0.043	65
