# Supplementary material for: TGF-ß Sma/Mab Signaling Mutations Uncouple Reproductive Aging from Somatic Aging
Source: PLoS Genet. 2009 Dec 24;5(12):e1000789. doi: 10.1371/journal.pgen.1000789 (PMC2791159; doi:10.1371/journal.pgen.1000789)
Supplement: Table S7 — Comparison of life spans and self-fertilized reproductive spans in mutants with extended reproductive spans. (0.10 MB PDF) [file pgen.1000789.s015.pdf]

Genotype	mean LS/RS± std. error	% change	P-value	N
**Life span experiment 1:**				
wild type	**15.8** ±0.4	**--**	--	72
sma-2(e502)	**18.2** ±0.7	**+15%**	0.0036	73
daf-7(e1372)	**21.1** ±0.6	**+34%**	<0.0001	73
**Life span experiment 2:**				
wild type	**12.7** ±0.5	**--**	--	69
daf-2(e1370)	**40.7**±2.5	**+220%**	<0.0001	70
eat-2(ad465)	**19.3** ±1.0	**+52%**	<0.0001	69
**Reproductive span experiment 1:**				
wild type	**3.9** ±0.3	**--**	--	12
sma-2(e502)	**8.7** ±0.8*	**+123%**	<0.0001	13
daf-7(e1372)	**5.6** ±0.2	**+44%**	<0.0001	17
**Reproductive span experiment 2:**				
wild type	**3.7** ±0.2*	**--**	--	30
daf-2(e1370)	**5.9** ±0.3	**+59%**	<0.0001	45
eat-2(ad465)	**8.2** ±0.4	**+122%**	<0.0001	44

*Corrected for matricide (animals bagged at the last day were assumed to have ceased its reproduction on that day to calculate the mean RS, to avoid underestimation due to high matricide rate). Such correction calculates a value closer to the true mean RS, and is important when comparing the relative increases in reproductive span.
