# Supplementary material for: TGF-ß Sma/Mab Signaling Mutations Uncouple Reproductive Aging from Somatic Aging
Source: PLoS Genet. 2009 Dec 24;5(12):e1000789. doi: 10.1371/journal.pgen.1000789 (PMC2791159; doi:10.1371/journal.pgen.1000789)
Supplement: Table S8 — Comparison of life spans and wild-type mated reproductive spans in mutants with extended reproductive spans. (0.08 MB PDF) [file pgen.1000789.s016.pdf]

Genotype	mean RS± std. error	% change	P-value	N=
**Life span**				
wild type	**17.6** ±0.6	**--**	--	72
sma-2(e502)	**18.4** ±0.3	**+5%**	0.52	96
daf-7(e1372)	**21.3** ±0.8	**+21%**	0.0001	97
daf-2(e1370)	**36.2** ±1.6	**+106%**	<0.0001	72
eat-2(ad465)	**21.3** ±0.9	**+21%**	0.0011	72
**Reproductive span (mated)**				
wild type	**6.6** ±0.4*	**--**	--	15
sma-2(e502)	**10.0** ±0.6*	**+52%**	0.0034	29
daf-7(e1372)	**7.8** ±0.2	**+18%**	0.04	39
daf-2(e1370)	**11.0** ±0.3*	**+67%**	<0.0001	55
eat-2(ad465)	**12.0** ±0.4*	**+82%**	<0.0001	69

*Corrected for matricide (animals bagged at the last day were assumed to have ceased its reproduction on that day to calculate the mean RS, to avoid underestimation due to high matricide rate). Such correction calculates a value closer to the true mean RS, and is important when comparing the relative increases in reproductive span.
