# Supplementary material for: TGF-ß Sma/Mab Signaling Mutations Uncouple Reproductive Aging from Somatic Aging
Source: PLoS Genet. 2009 Dec 24;5(12):e1000789. doi: 10.1371/journal.pgen.1000789 (PMC2791159; doi:10.1371/journal.pgen.1000789)
Supplement: Table S9 — Effects of daf-16 loss-of-function on life spans and reproductive spans in TGF-β Dauer and Sma/Mab mutants. (0.10 MB PDF) [file pgen.1000789.s017.pdf]

Genotype	mean LS/RS± std. error	% change	P-value	N=
**Life span experiment 1a:**				
daf-16(mu86);daf-7(e1372)	**13.2** ±0.2	--	--	72
daf-16(mu86)	**14.3** ±0.3	**+8%**	0.0004	95
daf-7(e1372)	**21.3** ±0.8	**+61%**	<0.0001	97
wild type	**17.6** ±0.6	**--**	--	72
**Life span experiment 1b:**				
daf-16(mu86);sma-2(e502)	**15.0** ±0.6	--	--	
daf-16(mu86)	**14.3** ±0.3	**-5%**	0.23	95
sma-2(e502)	**18.4** ±0.3	**+23%**	<0.0001	96
wild type	**17.6** ±0.6	**--**	--	72
**Life span experiment 2:**				
daf-16(mu86);sma-2(e502)	**16.6** ±0.4	--	--	71
daf-16(mu86)	**14.7** ±0.5	**-11%**	0.009	72
sma-2(e502)	**18.6** ±0.3	**+12%**	0.005	72
wild type	**17.0** ±0.4	**--**	--	72
**Reproductive span experiment 1:**				
daf-16(mu86);sma-2(e502)	**12.1** ±0.5	**--**	--	60
daf-16(mu86)	**3.5** ±0.2	**-71%**	<0.0001	30
sma-2(e502)	**10.0** ±0.3	**-17%**	0.12	60
**Reproductive span experiment 2:**				
daf-16(RNAi);sma-2(e502)	**8.8** ±0.4	**--**	--	50
wild type*;daf-16(RNAi)*	**3.5** ±0.1	**-60%**	<0.0001	30
sma-2(e502);control (RNAi)	**8.4** ±0.3	**-5%**	0.47	48
wild type*; control(RNAi)*	**3.8** ±0.2	**--**	--	30
**Reproductive span experiment 3:**				
daf-16(RNAi);dbl-1(nk3)	**5.6** ±0.2	**--**	--	60
wild type*;daf-16(RNAi)*	**3.4** ±0.1	**-39%**	<0.0001	30
dbl-1(nk3);control(RNAi)	**5.2** ±0.2	**-7%**	0.04	60
wild type*;control(RNAi)*	**3.7** ±0.1	**--**	--	30
**Reproductive span experiment 4:**				
daf-16(RNAi);sma-2(e502)	**6.5** ±0.2	--	--	26
sma-2(e502);control RNAi	**7.0** ±0.5	**+8%**	0.58	20
**Reproductive span experiment 5:**				
daf-16(mu86);daf-7(e1372)	**4.6** ±0.2	--	--	
daf-7(e1372)	**5.5** ±0.2	**+20%**	0.004	
daf-16(mu86)	**3.5** ±0.1	**-24%**		
**Reproductive span experiment 6a:**				
daf-16(mu86);sma-2(e502) mated	**9.7** ±0.4	--	--	30
sma-2(e502) mated	**10.0** ±0.6	**+3%**	0.42	29
daf-16(mu86) mated	**6.1** ±0.2	**-37%**	0.001	34
**Reproductive span experiment 6b:**				
daf-16(mu86);daf-7(e1372) mated	**6.7** ±0.4	--	--	44
daf-7(e1372) mated	**7.8** ±0.2	**+16%**	0.02	39
daf-16(mu86) mated	**6.1** ±0.2	**-9%**	0.13	34
