# Supplementary material for: TGF-ß Sma/Mab Signaling Mutations Uncouple Reproductive Aging from Somatic Aging
Source: PLoS Genet. 2009 Dec 24;5(12):e1000789. doi: 10.1371/journal.pgen.1000789 (PMC2791159; doi:10.1371/journal.pgen.1000789)
Supplement: Table S10 — Effects of pha-4 loss-of-function on life spans and reproductive spans in eat-2 and TGF-β Sma/Mab mutants. (0.10 MB PDF) [file pgen.1000789.s018.pdf]

Genotype	mean LS/RS± std. err.	Compared to:	% change	P-value	N=
**LS experiment:**					
wild type;*control(RNAi)*	**16.1** ±0.3	**--**	**--**	--	72
wild type;*pha-4(RNAi)*	**16.3** ±0.4	wild type;*control(RNAi)*	**+1%**	0.71	72
eat-2(ad465);control(RNAi)	**21.6** ±0.5	**--**	**--**	--	72
eat-2(ad465);pha-4(RNAi)	**18.6** ±0.5	*eat-2(ad465);control(RNAi)*	**-14%**	0.0004	72
sma-2(e502);control(RNAi)	**16.0** ±0.5	**--**	**--**	--	72
sma-2(e502);pha-4(RNAi)	**15.6** ±0.5	*sma-2(e502);control(RNAi)*	**-3%**	0.79	73
**Reproductive span experiment 1:**					
wild type;*control(RNAi)*	**4.2** ±0.2	**--**	**--**	--	30
wild type;*pha-4(RNAi)*	**3.4** ±0.1	wild type;*control(RNAi)*	**-19%**	0.02	30
eat-2(ad465);control RNAi	**6.5** ±0.3	**--**	**--**	--	60
eat-2(ad465);pha-4(RNAi)	**5.1** ±0.2	*eat-2(ad465);control(RNAi)*	**-22%**	0.0004	60
sma-2(e502);control(RNAi)	**9.9** ±0.6	**--**	**--**	--	60
sma-2(e502);pha-4(RNAi)	*	*sma-2(e502);control(RNAi)*	*	0.76	60
**Reproductive span experiment 2 (mated):**					
wild type;*control(RNAi)*	**6.3** ±0.2	**--**	**--**	--	61
wild type;*pha-4(RNAi)*	**6.4** ±0.2	wild type;*control(RNAi)*	**+2%**	0.84	66
eat-2(ad465);control(RNAi)	**10.0** ±0.2	**--**	**--**	--	114
eat-2(ad465);pha-4(RNAi)	**8.7** ±0.2	*eat-2(ad465);control(RNAi)*	**-13%**	0.0026	119
sma-2(e502);control(RNAi)	**8.3** ±0.2	**--**	**--**	--	112
sma-2(e502);pha-4(RNAi)	*	*sma-2(e502);control(RNAi)*	*	0.064	118

*Animals exhibit high matricide early in assay obscuring ability to measure true RS
