# Supplementary material for: TGF-ß Sma/Mab Signaling Mutations Uncouple Reproductive Aging from Somatic Aging
Source: PLoS Genet. 2009 Dec 24;5(12):e1000789. doi: 10.1371/journal.pgen.1000789 (PMC2791159; doi:10.1371/journal.pgen.1000789)
Supplement: Table S11 — Effects of dbl-1 over-expression on the reproductive span of eat-2 mutants. (0.07 MB PDF) [file pgen.1000789.s019.pdf]

Genotype	mean RS± std. err.	Compared to:	% change	P-value	N=
wild type	**4.2** ±0.3	wild type	**--**	--	16
eat-2(ad465)	**5.9** ±0.4	wild type	**+40%**	0.0019	29
dbl-1 OE	**3.9** ±0.2	wild type	**-7%**	0.4	27
eat-2(ad465);dbl-1 OE	**5.9** ±0.3	wild type	**+40%**	0.0001	29
		eat-2(ad465)	**0%**	0.83	
		dbl-1OE	**+51%**	<0.0001	
